# Multitasking across the lifespan in different task contexts

**DOI:** 10.1038/s41598-024-61859-w

**Published:** 2024-05-23

**Authors:** Nathan Van Humbeeck, Mira Van Wilderode, Reinhold Kliegl, Astrid van Wieringen, Ralf T. Krampe

**Affiliations:** 1 Brain & Cognition Group, University of Leuven (KU Leuven), Leuven, Belgium; 2 Research Group Experimental Oto-Rhino-Laryngology, University of Leuven (KU Leuven), Leuven, Belgium; 3 Division of Training and Movement Sciences, University of Potsdam, Potsdam, Germany; 4 Dept of Special Needs Education, University of Oslo, Oslo, Norway

**Keywords:** Human behaviour, Geriatrics, Paediatrics

## Abstract

We assessed lifespan development of multitasking in a sample of 187 individuals aged 8–82 years. Participants performed a visuo-spatial working memory (VSWM) task together with either postural control or reaction time (RT) tasks. Using criterion-referenced testing we individually adjusted difficulty levels for the VSWM task to control for single-task differences. Age-differences in single-task performances followed U-shaped patterns with young adults outperforming children and older adults. Multitasking manipulations yielded robust performance decrements in VSWM, postural control and RT tasks. Presumably due to our adjustment of VSWM challenges, costs in this task were small and similar across age groups suggesting that age-differential costs found in earlier studies largely reflected differences already present during single-task performance. Age-differences in multitasking costs for concurrent tasks depended on specific combinations. For VSWM and RT task combinations increases in RT were the smallest for children but pronounced in adults highlighting the role of cognitive control processes. Stabilogram diffusion analysis of postural control demonstrated that long-term control mechanisms were affected by concurrent VSWM demands. This interference was pronounced in older adults supporting concepts of compensation or increased cognitive involvement in sensorimotor processes at older age. Our study demonstrates how a lifespan approach can delineate the explanatory scope of models of human multitasking.

## Introduction

Receiving an unexpected phone call in the midst of preparing for an upcoming meeting is commonly perceived as disruptive and potentially damaging to one’s performance. Continuous sensorimotor tasks like walking or standing, on the other hand, are rarely considered detrimental for ongoing cognitive processes because they are presumably automatic or, at the very least, can be put on autopilot temporarily. While both examples constitute instances of multitasking, their challenges have not been compared within the same study. Likewise, theories of multitasking do not address differences between coordinating cognitive tasks with either continuous sensorimotor challenges or tasks demanding immediate attention (i.e., switching).

Extant theories attribute multitasking costs to task competition for overall resources and their reduced availability in later adulthood^1^. A second class of models emphasizes domain-general functions like cognitive control as critical mechanisms allocating resources among concurrent tasks, switching among them, and inhibiting mutual task interference^2^. Regarding developmental constraints, both approaches assume similar limitations at the two ends of the lifespan with resources and executive functions either not fully matured or declined compared with young adults (i.e., U-shaped pattern of multitasking performance across lifespan development)^3–5^. However, studies on multitasking with children do not support this U-shape assumption but showed a mix of pronounced costs, no costs, or even dual-task improvements^6–11^. In addition, evidence on multitasking in older age is not unequivocal either^12^. Arguably, these inconsistencies in the literature arise from the key methodological problem of designing tasks which provide comparable single-task challenges to different age groups, avoiding floor- and ceiling effects^6,13^. In the present study, participants between 8 and 81 years of age performed a VSWM task, individually adjusted to provide comparable challenges between age groups, while concurrently performing a continuous sensorimotor task (postural control) or a task requiring immediate attention and switching (a simple reaction time task [RT]).

The view that continuous sensorimotor tasks like postural control do not demand cognitive processing resources may be overly optimistic because simply maintaining a stable posture in upright stance already presents enough of a challenge to interfere with concurrent cognitive tasks, at least in older adults^14,15^. The study of age-differences in cognition-posture multitasking was pioneered by Shumway-Cook and her colleagues. In an early study they had healthy young adults and older adults with and without balance impairment perform quiet standing in isolation as well as in combination with a cognitive task^4^. They hypothesized that age-related alterations in balance control (e.g., deterioration of sensory systems^16^, decreasing muscle strength^17^) require older adults to allocate more cognitive resources towards postural control when concurrently performing a cognitive task. Their results did not support this “posture first” hypothesis. However, later studies did find postural prioritization in older adults in conditions of increased challenges to stability^18–20^.

Boisgontier et al. concluded from their review that most studies point towards higher costs in older compared with young adults when cognitive and posture tasks had to be performed concurrently, although this evidence is far from unequivocal^14^. For example, Bock^12^ analyzed data from experiments combining walking with a wide range of cognitive tasks, revealing that age-related difficulties in dual-tasking were not consistent across tasks. It was suggested that the nature of the cognitive task plays a critical role in the degree to which adult age-differences explain dual-task costs, with tasks requiring high visual demands showing more multitasking interference with increasing age^12^.

A similar pattern of unequivocal evidence emerges when cognitive-posture multitasking interference at the other end of the lifespan is considered. While some evidence points towards higher levels of combined interference in children^21,22^, several studies found either no or reduced levels of interference^7–10,23^. For example, Schaefer et al. used a combination of wobbleboard-balancing and memorizing tasks to compare multitasking of 9- and 11-year old children with young adults^9^. Young adults did show dual-task decrements in both cognitive and posture tasks. However, children showed only cognitive dual-task costs and improved their balance under dual-task conditions. A later study by Pavão et al. with children, adolescents and young adults found similar results^23^. Here, children showed the smallest dual-task postural sway increases when comparing single to dual-task performance.

In a study by Krampe et al., multitasking performance across the lifespan was directly studied by comparing children, young, and older adults who simultaneously engaged in walking and cognitive tasks^24^. Results showed a U-shape relation between age and dual-task costs for the walking task. For the concurrent cognitive task, children showed the highest costs, while costs in young and older adults were similar and not reliably different from zero. The notion of lifespan changes in general processing resources determining a U-Shape development of multitasking corresponds to data from fluid-intelligence functions like processing speed, WM span^25^, simple reaction times^26^ or postural stability^27^.

The general resource model of multitasking argues that the factors and constraints determining single- and dual-task performance are the same (i.e., all tasks draw from the same finite pool of general resources). In line with this claim, Krampe et al. found that single-task performance accounted for 55% of the variance in dual-task performance in their lifespan sample^24^. However, reliable age-related variance remained in the residuals because the model systematically overestimated children’s and underestimated young adults' performances.

When assuming limitations in single-task performance are a key factor in explaining age-related dual-task differences, the question arises whether age-differential dual-task patterns persist when accounting for individual disparities in single-task performance. That is, the same cognitive single-task (e.g., serial sevens) may be too difficult for children and old adults and too easy for young adults. Therefore, excess dual-task decrements in "weaker" groups might reflect higher resource demands for single-task cognitive performance instead of higher multitasking interference. A method avoiding some of these pitfalls is to individually calibrate difficulties for the cognitive task. Anderson et al. designed a multitasking paradigm in which the individual tasks (digit span, inspection task) were titrated to ensure that any age-differential dual-task costs could not be ascribed to differences in single-task performance^6^. While results showed robust dual-task decrements, they did not show an age-differential pattern, highlighting the importance of individually adjusted single-task conditions.

More recent models focused mechanisms of resource allocation like cognitive control (executive functions)^28–30^. Neuroimaging studies have identified increased activations in specific regions like the anterior cingulate cortex or inferior frontal junction when multiple dimensions must be attended or continuous switching among tasks is required^31^. An implication of this approach is that multitasking interference should depend on the degree to which component tasks themselves rely on these domain-general processes. This was indeed observed in several studies^32,33^. Cognitive control accounts of multitasking are not mutually exclusive with resource accounts, and considering developmental changes in cognitive control, they predict similar U-shape lifespan trajectories for multitasking.

Several authors argued that motor tasks and postural control in particular pose special challenges for older adults. Neuroimaging studies found more extensive cortical activation for motor tasks in later adulthood which the authors interpreted as evidence for decreased automatization of movement control in the elderly^34–36^. The cognitive compensation hypothesis proposed by Li and Lindenberger argued that older adults permanently invest cognitive processing capacity to compensate for declines in sensorimotor functions^37^. As a result, combinations of cognitive and postural control tasks yield higher performance decrements in the elderly when compared with younger adults.

The inconclusive picture arising from age-comparative research has made it hard to disambiguate accounts of multitasking. Apart from individual differences with regard to the difficulty of the cognitive task, another potential account for the lack of conclusive age-differential findings in cognitive-posture multitasking is the utilization of traditional variables quantifying center-of-pressure (CoP) movement based on assumptions of linearity (e.g., total displacement, mean sway velocity), stationarity (e.g., ellipse area, sway variability), or both. Linearity assumptions treat all sway (e.g., COP-movement) as regardless of whether it occurs near to boundaries of stability or well within the center of our stability limits. Measures presuming stationarity consider sway to occur around a single reference point, and they cannot distinguish between someone requiring constant postural corrections and someone slowly drifting away from their initial position^27^.

Collins and De Luca pioneered a method of quantifying postural control which addresses these problems^38^. Stabilogram diffusion analysis (SDA) adopts Einstein's approach to Brownian molecular motion and applies it to postural control. The basic approach involves averaging the squared displacement of all measurement points of a time series as a function of its time interval. Such modeling reveals postural control processes at different timescales: an initial steep increase in displacement at time intervals typically shorter than 1 s (short-term diffusion) and a much shallower increase for longer delays (long-term diffusion). Short-term diffusion reflects persistent displacement which is plausibly linked to biomechanical factors (stiffness, anthropometric characteristics) and continuous control mechanisms (feedforward strategies and reflex responses). On the other hand, long-term diffusion reflects anti-persistent displacement which is thought to be determined by the position-based consistency of feedback time-delayed postural adjustments. Two additional parameters which can be derived from SDAs are the critical delay and the critical displacement, respectively reflecting the timing (delay) and displacement of transition between short- and long-term diffusion processes.

Van Humbeeck et al. refined the SDA method and applied it to data from a lifespan sample of children, young, and older adults who performed different postural control tasks^27^. Traditional summary statistics (pathlength, COP-ellipse area) produced the expected U-shaped lifespan trajectory with similar levels of performances in children and older adults. However, SDA modeling revealed that these similarities at the levels of gross performances resulted from developmental differences in component processes. Older adults showed pronounced short-term diffusion (especially under more challenging conditions) compared with children but could largely compensate for this through adequate corrective responses (long-term diffusion) which was almost at young-adult levels. Different from an earlier proposal by Collins and De Luca, Van Humbeeck et al. found empirical support for Peterka’s claim that the critical delay was far from a passive reflection of time demands for multisensory processes, but was adapted to postural control challenges, particularly by older adults^27,38,39^.

The current study tested the effects of lifespan development on multitasking costs under conditions where cognitive single-task demands were individually adjusted. To this end, we combined a VSWM task with either a postural control or RT task. The RT-VSWM combination served as a control to determine whether age-differences in multitasking we might observe were specific to sensorimotor-cognitive combinations or generalized to other task combinations. In particular, we aimed to come up with a simple secondary task unlikely to interfere with processes specific to the VSWM task but mainly challenge immediate switching to, and prioritization of, the RT task. It is in this spirit that we designed the second task combination and interpreted related results.

From the general resource account, a smaller pool of resources at both ends of the lifespan predicts similar patterns of increased interference in children and older adults compared with young adults in both VSWM-RT and VSWM-posture multitasking settings. However, in line with cognitive compensation, that is attributing a special status to postural control for older adults, we predict more interference in the VSWM-posture than the VSWM-RT multitasking setting for older adults. Finally, in line with cognitive control, we expected larger costs for the VSWM-RT than the VSWM-posture multitasking combination. The reason is the necessity for immediate task switches in the former task combination, with similar patterns of age-related differences.

Focusing on VSWM-posture multitasking, we aimed to identify the SDA parameters and associated component processes affected by concurrent cognitive demands. Based on previous findings, the component processes we consider most likely affected by concurrent task performance are the timing and quality of postural adjustments reflected in the critical time interval and long-term diffusion, as both of these parameters have shown high levels of adaptability in contexts where task difficulty was manipulated^27^.

We aimed to extend the findings by Van Humbeeck et al. by first, distinguishing between anterior–posterior (AP)- and medio-lateral (ML) sway and second, by including middle-aged adults. The separation of AP and ML sway was motivated by recent studies indicating that during childhood and adolescence control of AP-sway proceeds at a faster rate and reaches adult-levels at an earlier age than ML-sway^40,41^. Likewise, studies suggest that control of ML-sway is more important for older adults because recovery from ML instability is more difficult than for AP-sway, and ML-sway has been associated to increased fall risk in older adults^42–45^. Inclusion of middle-aged adults allows us to further evaluate our earlier hypothesis with regard to increased adaptability of critical time intervals and long-term diffusion. Here, we hypothesize that middle-aged adults can at least partially compensate for age-related alterations presumably affecting short-term stability by adjusting the timing and quality of long-term control processes.

## Results

**Figure 1:**
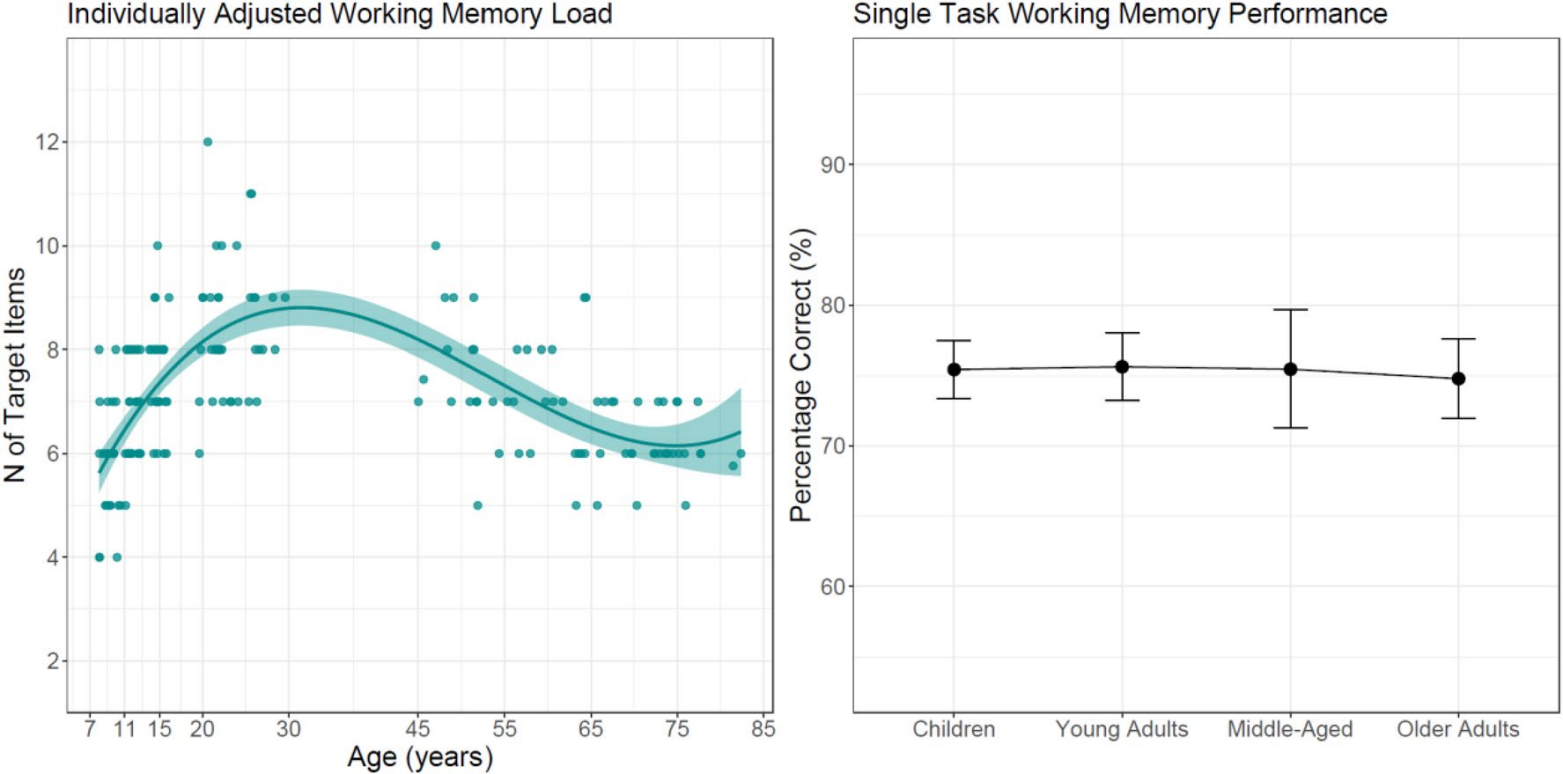
Number of target items presented in the working memory task (left panel) to attain comparable single-task percentages correct across age groups (right panel). Error envelope (left) represents 1 standard error, error bars (right) represent 95% between-group confidence intervals.

Figure 1 depicts individually adjusted VSWM load (left panel) that is, the number of target items presented in each trial determined by criterion-referenced testing. As expected, this variable showed an inverted J-shaped function of age, with young adults outperforming children (β = 1.77, SE = 0.214, t = 8.253, p < 0.001) and pooled middle-aged and older adult groups (β = 1.131, SE = 0.147, t = 7.682, p < 0.001). Middle-aged adults had higher scores than older adults (β = 0.482, SE = 0.136, t = 3.560, p < 0.001). Nested effects of age reflected developmental gains in children (β = 0.284, SE = 0.051, t = 5.600, p < 0.001) and age-related declines in middle-aged adults (β = − 0.073, SE = 0.029, t = 2.543, p = 0.015). A one-way ANOVA indicated no differences in percentage correct scores between age groups under single-task conditions (p = 0.944).

**Figure 2:**
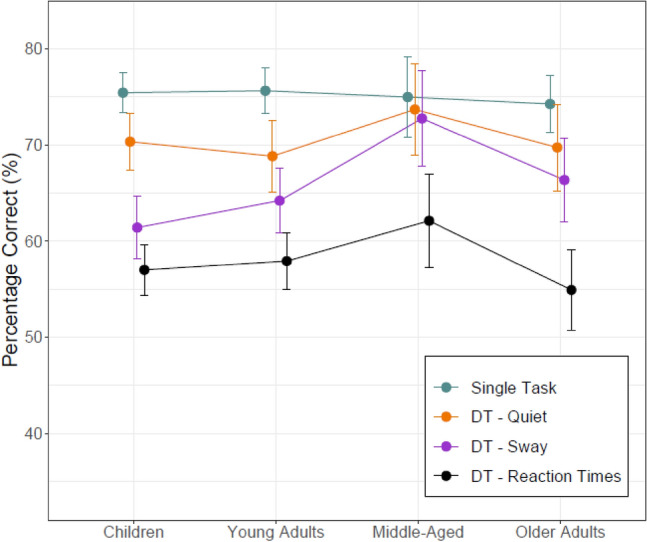
Working memory performance in single- and three dual-task conditions (DT—Quiet, DT—Sway, DT—Reaction Time). Error bars represent 95% between-group confidence intervals.

Figure 2 shows that all multitasking manipulations were effective in significantly reducing VSWM performances. Percentage correct scores were significantly lower under concurrent postural control demands for combined (quiet + sway-referenced) dual-task conditions than single-task conditions (z = 9.045, p < 0.001). With the exception of middle-aged adults (t = 1.370, p = 0.179), this pattern generalized to all other age groups (t’s > 3.580, p < 0.001). Performance reductions were also larger for sway-referenced than quiet-stance conditions (z = 4.202, p < 0.001). That is, there is evidence that multitasking costs in VSWM reflected the difficulty of the posture task. This effect was significant in children (t = 4.998, p < 0.001) and young adults (t = 2.242, p = 0.031). This effect was also larger for young than pooled older adults (z = 2.961, p < 0.001, for the group x condition interaction). However, upon closer inspection this interaction was solely due to middle-aged adults who apparently maintained their cognitive performances irrespective of balance challenges. Multitasking costs imposed by the concurrent RT task were significant (z = 16.877, p < 0.001) overall and in all age groups (t’s > 6.920, p < 0.001).

**Figure 3:**
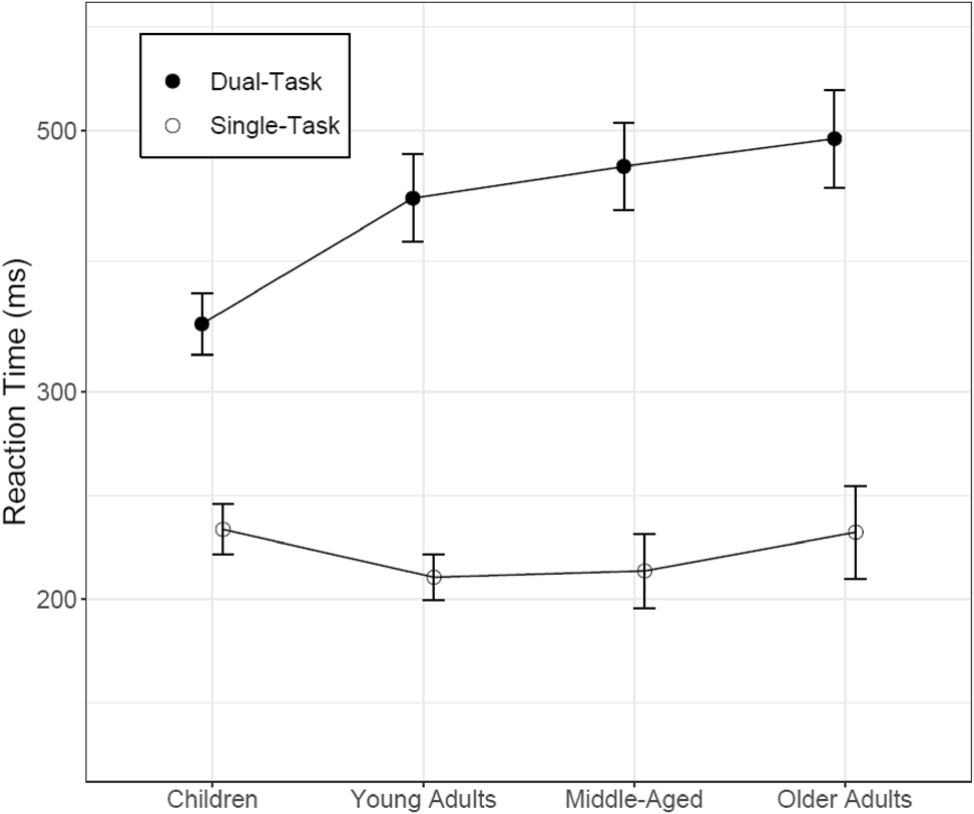
Reaction times under single-task and dual-task conditions for all age groups. Error bars represent between-group confidence intervals.

Figure 3 illustrates that concurrent VSWM demands led to significant increases in RT (z = 39.401, p < 0.001), and this was true for all age groups (t’s > 16.039, p < 0.001). There was no evidence for significant differences between adult-age groups. However, children responded slower under single-task but faster than young adults under dual-task conditions, yielding a reliable age group effect (z = 2.091, p = 0.0380) which was qualified by a context by age group interaction (z = 7.924, p < 0.001). Nested effects of age revealed faster RTs with increasing age in children (z = 4.580, p < 0.001) with larger developmental gain in the single-task (z = 2.843, p = 0.005, for context x age group interaction). In contrast, older adults' RTs were similar to children's under single-task conditions, but reliably slower when concurrent working memory demands had to be accommodated (z = 7.714, p < 0.001, for the interaction between context and the direct comparison of older adults and children).

**Figure 4:**
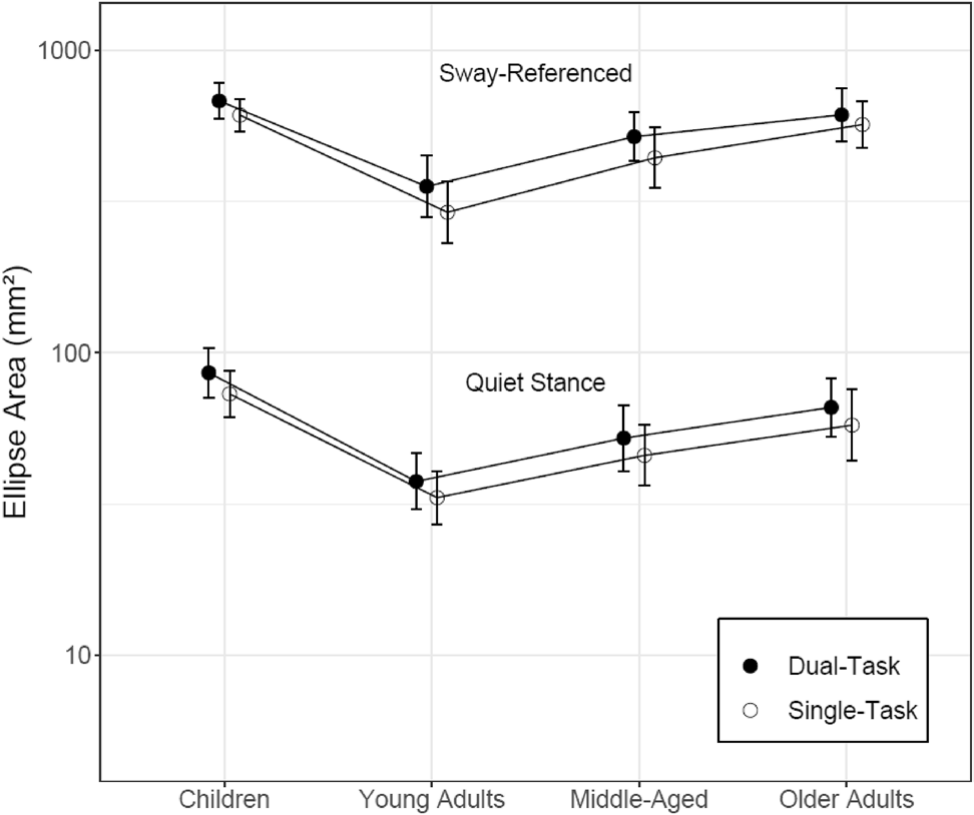
Postural control performance according to the CoP ellipse area under single- and dual-task context for side-by-side quiet and sway-referenced conditions in all age groups. The CoP ellipse area was determined by fitting an ellipse spanning 1.96 standard deviations (95% of datapoints) of the principle axes of the CoP trajectory (see Oliveira et al. 1996). Error bars represent 95% between-group confidence intervals.

Figure 4 illustrates the effects of task difficulty and multitasking on postural control for the COP ellipse area, a widely used summary statistic. Not surprisingly, areas were smaller for the side-by-side quiet compared with the sway-referenced condition (t = 47.173, p < 0.001). Compared with single-task conditions CoP areas increased significantly when participants performed the VSWM task (z = 4.944, p < 0.001), however increases were similar across age groups. As expected, young adults outperformed children (z = 7.567, p < 0.001) and pooled older adults (z = 4.600, p < 0.001), while children performed at levels comparable to older adults (z = 1.724, p = 0.086). Nested effects of exact age indicated age-related performance improvements in children (z = 4.165, p < 0.001).

**Figure 5:**
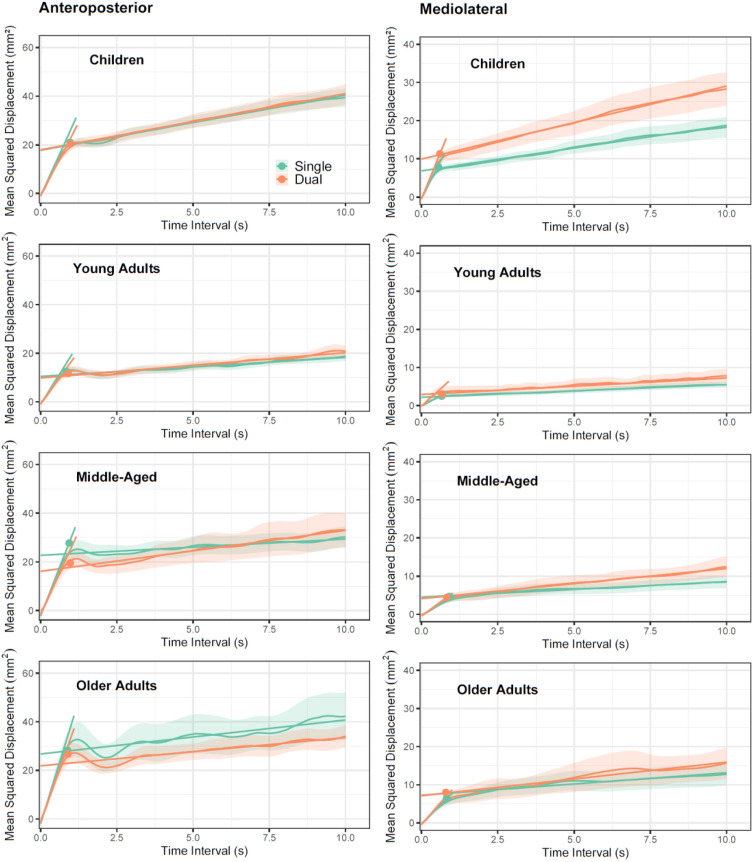
Averaged stabilogram diffusion plots for AP (left) and ML (right) COP sway with fit lines based on parameter estimates for short- and long-term diffusion coefficients in children, young, middle-aged and older adults in side-by-side quiet stance. Symbols denote the critical point. Error envelope =  ± 1SE.

To determine how component processes of postural control were affected by multitasking we applied SDA to quiet-stance conditions data. Considering their different developmental trajectories, we conducted separate analyses for AP and ML displacements. Figure 5 shows SDA plots for age groups in AP (left column) and ML (right column). Group means for the five parameters are shown in Table 1. The component postural control processes we had considered most likely candidates were indeed affected by concurrent working memory demands. AP long-term diffusion coefficients were higher under dual-task compared with single-task conditions (z = 2.749, p = 0.006) and critical time intervals for onset of AP-corrections were longer during multitasking (z = 2.004, p = 0.047).

**Table 1:** Postural control measures for single- and dual-task contexts in all age groups.

		Children		Young adults		Middle-aged adults		Older adults	
Measure	Context	M	SD	M	SD	M	SD	M	SD
Ellipse area (mm^2^)	Single task	95.79	74.02	40.53	31.75	59.08	43.07	90.40	97.19
	Dual task	115.92	91.92	48.52	47.31	70.10	59.65	81.40	53.36
AP short-term diffusion coefficient (mm^2^ s^−1^)	Single task	10.87	7.41	8.27	7.02	13.24	10.30	17.75	21.31
	Dual task	10.62	7.46	7.86	8.78	12.05	15.07	14.95	11.63
AP long-term diffusion coefficient (mm^2^ s^−1^)	Single task	0.66	0.75	0.27	0.20	0.21	0.26	0.53	1.32
	Dual task	0.70	1.07	0.40	0.33	0.72	1.88	0.34	0.39
AP critical time interval (s)	Single task	0.91	0.22	0.82	0.26	0.92	0.32	0.88	0.32
	Dual task	0.96	0.30	0.87	0.21	0.95	0.27	0.87	0.27
AP critical mean squared displacement (mm^2^)	Single task	17.81	10.45	11.57	8.66	23.00	22.90	33.40	53.97
	Dual task	19.44	16.42	11.84	11.43	19.34	18.57	24.63	19.83
ML short-term diffusion coefficient (mm^2^ s^−1^)	Single task	6.48	5.30	1.99	1.84	1.95	1.45	3.36	3.06
	Dual task	7.53	7.39	2.72	6.13	2.50	2.81	3.62	3.20
ML long-term diffusion coefficient (mm^2^ s^−1^)	Single task	0.37	0.50	0.13	0.09	0.12	0.15	0.13	0.15
	Dual task	0.55	0.70	0.18	0.21	0.31	0.41	0.26	0.26
ML critical time interval (s)	Single task	0.54	0.24	0.67	0.34	0.88	0.33	0.84	0.37
	Dual task	0.57	0.24	0.67	0.24	0.81	0.31	0.79	0.34
ML critical mean squared displacement (mm^2^)	Single task	6.38	6.47	2.63	3.46	3.56	3.89	5.53	5.54
	Dual task	8.80	11.89	3.25	5.83	3.90	5.27	5.43	5.78

Note that these values do not depict the transformed measures used for statistical analysis.

*M* mean, *SD* standard deviation.

While AP effects were similar across groups, context effects in ML-displacement showed marked age-differential patterns. ML long-term diffusion systematically increased under dual-task conditions (z = 5.555, p < 0.001), with pronounced effects in pooled middle-aged and older compared with young adults (t = 2.105, p = 0.036). Post-hoc tests confirmed robust increases in all age groups (t’s > 2.830, p’s < 0.007) except for young adults (t = 1.307, p = 0.199). Children increased critical intervals, while older adults shortened the time for transitioning between control processes (Table 1; z = 2.279, p = 0.023 for context x age group interaction) under dual-task conditions. Compared with young adults, the pooled older adults also robustly shortened their transitioning time (z = 2.190, p = 0.029). Post-hoc tests showed significant multitasking effects indicating larger ML critical time intervals in children under dual-task conditions (t = 2.320, p = 0.023).

Somewhat unexpected, we also found a main effect of context for short-term AP-diffusion (z = 2.035, p = 0.043). Post-hoc comparisons revealed that middle-aged adults had reliably shallower short-term diffusion slopes in dual- compared with single-task conditions (t = 2.101, p = 0.043).

In addition to effects associated with multitasking, the overall pattern of group differences for parameters confirmed the hypothesized U-shape with young adults having smaller short- as well as long-term diffusions and critical displacement than children (respectively in AP: z = 2.993, p = 0.003; z = 2.853, p = 0.005; z = 4.199, p < 0.001) (respectively in ML: z = 8.748, p < 0.001; z = 5.192 p < 0.001; z = 6.075, p < 0.001) on the one hand and pooled older adults on the other (respectively in AP: z = 4.178, p < 0.001; NS; z = 4.840 p < 0.001) (respectively in ML: NS; NS; z = 2.786, p = 0.006). Likewise, young adults' critical time intervals tended to be shorter than in the other groups. Age effects concerning children were much larger for ML than for AP-displacement supporting our distinction of related parameters and this was also true for changes between middle- and later adulthood. When directly compared children's overall performance resembled that of older adults.

One important qualification does not align with these general findings. While children displayed the longest critical time intervals in the AP direction, they actually reduced critical time intervals in the ML direction when compared with young adults (z = 2.970, p = 0.003) and older adults (z = 5.247, p < 0.001).

**Figure 6:**
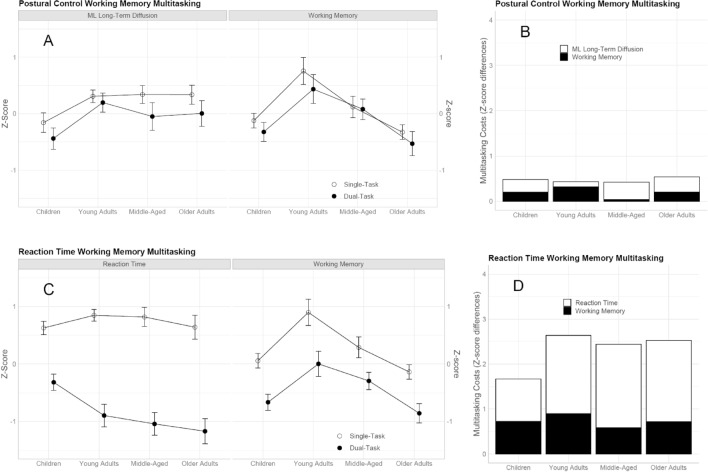
Z-scores for single- and dual-task performances in both multitasking settings (**A**,**C**) and their combined dual-task costs (**B**,**D**). For panel A and C, higher scores indicate better performances. For panel B and D, higher scores indicate larger dual-task decrements. Error bars represent 95% between-group confidence intervals.

To compare multitasking performance between tasks and assess differences in overall costs of multitasking, we calculated z-transformed performance measures in each of the task combinations (Fig. 6A,C) as well as their absolute costs depicted as stacked columns (Fig. 6B,D). To emphasize group differences related to the absolute performance in the VSWM task, we z-transformed the total score (score per target * number of target items), rather than percentage correct which does not take into account the number of target items. For postural control we included medio-lateral long-term diffusion as this measure shows to be the most sensitive to age-differential effects of multitasking in our study.

In line with analyses reported earlier, the posture-VSWM model showed a reliable effect of task context (z = 7.570, p < 0.001), indicating lower levels of performance under concurrent task demands. Likewise, young adults performed better than children (z = 8.850, p < 0.001) as well as pooled middle-aged and older adults (z = 5.303, p < 0.001), and middle-aged adults outperformed older adults (z = 2.550, p = 0.012). Several novel findings emerged from this analysis. A measure by age group interaction revealed disproportionately better working memory compared with postural control performances in young compared with pooled older adults (z = 4.478, p < 0.001). When compared with older adults, middle-aged adults also performed disproportionately better in working memory when compared with postural control (z = 3.087, p = 0.002). A robust three-way age group by context by measure interaction immerged when comparing young adults with children (z = 2.076, p = 0.038) and the pooled older adults (z = 3.011, p = 0.003). Post-hoc t-tests indicated that, in contrast to the other age groups, postural control did not show robust context effects in young adults (t = 1.131, p = 0.199). Also, in contrast to the other age groups, working memory did not show robust context effects in pooled older adults (t = 1.96, p = 0.055). Nested effects of age showed age-related performance improvements in children (z = 5.214, p < 0.001). A measure by nested age interaction indicated larger performance decrements in the working memory when compared with the postural control task in middle-aged adults.

Analysis for the VSWM-RT multitasking setting replicated overall effects of task context (z = 46.564, p < 0.001) and this was true for the main differences among age groups: overall, young adults outperformed the children (z = 4.354, p < 0.001) and pooled older adults (z = 6.315, p < 0.001); middle-aged showed better performance when compared with older adults (z = 4.008, p < 0.001). A measure by context interaction revealed larger dual-task decrements in the RT task when compared with the working memory task (z = 17.278, p < 0.001). Apart from replication of previously reported findings, this LMM showed a measure by group interaction indicating disproportionately better performance on the working memory when compared with the RT task for the young adults when compared with the children (z = 7.615, p < 0.001) and the pooled older adults (z = 4.322, p < 0.001). Relative to the older adults, middle-aged adults’ performance was disproportionately better on the working memory task when compared with the RT task (z = 2.356, p = 0.020). A context by group interaction revealed pronounced context effects in the young adults when compared with the children (z = 7.871, p < 0.001). A three-way interaction indicated pronounced dual-task decrements in young adults when compared with children for the RT task but not in the working memory task (z = 5.010, p < 0.001). Further, when compared with pooled older adults, young adults showed pronounced dual-task decrements in the working memory task when compared with the RT task (z = 2.624, p = 0.009).

Multitasking costs differed vastly between multitasking settings; higher multitasking costs were present in the VSWM-RT combination when compared with VSWM-posture (z = 22.169, p < 0.001). In general, the concurrent tasks (RT/postural control) showed more multitasking costs when compared with the VSWM task (z = 11.393, p < 0.001). A main effect of age group between children and young adults was qualified by an interaction with multitasking setting, revealing fewer costs in the children group which was only robust in the VSWM-RT setting (z = 4.913, p < 0.001). A task by age group interaction (z = 3.727, p < 0.001) revealed disproportionately larger costs in young adults for the VSWM but not for the concurrent task when compared with the pooled older adults. The difference between the concurrent task and the VSWM task was dependent on the multitasking setting, and this was indicated by a setting by task interaction revealing larger differences in multitasking costs between RT and VSWM than between postural control and VSWM (z = 9.252, p = 0.001). A three-way interaction indicated that this difference was not of the same magnitude in children and young adults, suggesting differences are larger in young adults when compared with children (z = 4.382 p < 0.001).

## Discussion

Our study is the first to investigate multitasking in a lifespan sample using two different task combinations. To this end we combined a visuo-spatial working memory (VSWM) task on the one hand with reaction time (RT) and postural control tasks on the other. By means of criterion-referenced testing we individually calibrated VSWM task demands, successfully equating age groups at the level of percentages correct in single-task contexts. This methodological approach was key for our goal to avoid ceiling- or floor effects which presumably disguised dual-task effects in young adults and overestimated them in children or older adult groups in previous studies^6^.

Calibration data for working memory and single-task performances in posture and RT tasks revealed the typical U-shape function with young adults outperforming all other groups and children performing at comparable levels with older adults. Applying stabilogram-diffusion analysis (SDA) enabled us to replicate the developmental changes in control processes shown in Van Humbeeck et al. (2023). Two novel findings emerged: first, our results revealed that developmental trajectories for postural control of AP- and ML-sway differ considerably between age groups. Pronounced effects in children when ML-sway was considered confirm earlier hypotheses that related control processes lag behind AP-control during development^40^. Second, markedly different behavior in long-term control processes supported the idea that middle-aged adults could compensate for age-related alterations by adjusting long-term control processes.

Our multitasking manipulations yielded robust performance decrements for all task combinations. Increasing postural control challenges directly affected concurrent VSWM performance in most groups thereby demonstrating that to a certain extent, related tasks share common underlying mechanisms. Different from several earlier studies we did not see pronounced multitasking costs in the cognitive task for children nor older adults^7,15,19^. We attribute this to our criterion-referenced testing approach. Here we follow Anderson et al. who found similar results combining two cognitive tasks and argued that age-differential dual-task costs at both ends of the lifespan partially reflect limitations in single-task performance^6^. A noteworthy exception from the overall pattern were middle-aged adults who retained single-task VSWM performance.

We did find age-differential costs of multi-tasking in postural stability, however. SDA parameters revealed that long-term control and the relative timing of postural control mechanisms were affected by concurrent working memory demands. Importantly, these effects were pronounced in middle-aged and older adults compared with young adults. This finding is in line with the cognitive compensation hypothesis suggesting that middle-aged and older adults recruit "cognitive" processes to a larger degree than young adults when controlling their posture^37^. Age-differential effects showed for ML- but not for AP-sway, signifying that the sway axis linked to an elevated risk of falls in the elderly is also the most responsive to multitasking in this age group^42,44,45^. Note that traditional summary measures of postural stability like the ellipse area failed to discover age-differential performance decrements under dual-task conditions pointing to a possible source of inconsistent findings in previous studies.

We had argued that the VSWM-RT task combination provided larger challenges compared with the postural control task because responses to auditory stimuli demanded instant task switching leaving little room for individual resource allocation strategies. In line with these assumptions, we found higher multitasking costs in this setting, and this was true for all age groups. We also found age-differential costs, however, the pattern differed markedly from the VSWM-posture setting. Adult age groups showed comparable declines under dual-task conditions while children showed the smallest effects of all groups with significantly smaller decrements in the RT task. In interpreting these findings, it is important to consider that children (like older adults) performed the VSWM task with fewer visual stimuli than young adults. Thus, they encountered fewer instances of overlapping sensory input (i.e., an auditory stimulus interrupting encoding of a VSWM task target) and related task-switches. Attesting young adults more cognitive resources and superior task-switching abilities compared to children, this result clearly shows limitations of multitasking in the high-performance group of young adults. The key question remains why children, but not older adults, were able to benefit from the advantage of facing fewer VSWM targets? The different patterns for the two multitasking settings are not easily reconciled with resource accounts and they also go beyond the explanatory scope of the cognitive compensation hypothesis in our view. We argue that age-specific changes in cognitive-control mechanisms must be considered in this context. Previous studies uncovered advantages for children compared with older adults related to superior inhibitory control^46^. The concept of inhibition is being used to account for a variety of neurocognitive behaviors. Here we follow Miyake and colleagues who argued that inhibition constitutes a general executive function ability relevant to task-switching as well as working memory updating^47^. Specific adult-age task-switching deficits have been demonstrated for the maintenance of task sets^48^ and the inhibition of previously used task-sets^49^.

Limitations of our study concern the SDA methodology which could only be applied to quiet-stance posture not be extended to sway-referenced conditions. Another weakness of this manipulation was that it was obviously too difficult for many older adults. Future studies should use difficulty manipulations suitable for all types of analyses and participants such as (semi-)tandem stance. Much of our reasoning about differences between task combinations is obviously post-hoc and in hindsight independent assessments of cognitive control processes like switching or inhibition would have been an advantage.

In conclusion, we believe that our study goes a long way in replacing the intuitive but circular "resources" in traditional approaches with specific mechanisms affected by multitasking. We also showed how timing and quality of long-term postural control mechanisms play specific roles in different age groups and their adaptation to multitasking challenges. Different task combinations tax cognitive control mechanisms to various degrees with consequences depending on the age group under consideration. Our study provides a blueprint for combining individually adjusted testing and LMMs to disambiguate among theoretical models of multitasking. Though real-life does not provide for individually calibrated challenges, we believe our research provides insights into how vulnerable groups navigate daily life multitasking situations.

## Methods

### Participants

A total of 75 children (8 to 15 years of age), 38 young adults (20 to 29 years of age), 37 middle-aged adults (45 to 65 years of age), and 37 older adults (66 to 82 years of age) were recruited through word of mouth advertisement and contacts with local schools. We did not include participants who had (a) a recent fall or sports accident, (b) a history of neurological disorders or stroke, (c) a neurodevelopmental disorder known to affect balance (i.e., ADHD), (d) signs of cognitive impairment, (e) an acute musculoskeletal injury or (f) consumed alcohol within 24 h before the experiment. Screening of cognitive impairment included a modified version of the Cognitive Disorders Examination^50^. Persons categorized as having a very high probability of dementia (i.e., 1 middle-aged and 8 older adults) were excluded from data analysis. Between blocks of experimental trials, cognitive abilities were assessed using the digit symbol substitution and the digit span^51^. Sample characteristics of participants included in the data analysis are provided in Table 2. Individual test sessions lasted between 1.5 and 2 h. Participants received the equivalent of €8 per hour for participation. The study was conducted in accordance with the ethical standards laid down in the Declaration of Helsinki and approved by the Medical Ethics Committee (KU Leuven/UZ Leuven). Informed consent was obtained from all participants. Informed consent was signed by parents in the case of children and adolescents.

**Table 2:** Descriptive characteristics of each age group.

	Age	SD age	N	Sex		DSF	DSB	DSS
				Male	Female			
Children (8–16 yo)	11.9	2.4	75	37	38	8.6	5.8	59.8
Young adults (19–30 yo)	23.3	2.7	38	19	19	10.9	8.3	93.4
Middle-aged adults (45–65 yo)	56.1	6.3	36	19	17	9.6	7.8	82.6
Older adults (66–82 yo)	73.2	4.0	30	13	17	9.4	6.4	65.4

*DSF* digit span forward, *DSB* digit span backward, *DSS* digit symbol substitution.

### Tasks

The VSWM task consisted of an encoding and retrieval phase. During the encoding phase, participants were required to monitor visual stimuli (red apples) which successively appeared for 400 ms durations in one of 12 boxes arranged in a 3 × 4 grid^52^ on a 15.6″ Dell laptop screen. Different items (i.e., house, car, train, sailboat) and colors (i.e., blue, green, black) identified the columns and rows of the grid respectively. This design yielded a unique pairing of item and color which was displayed above each individual box. Successive apples occurred at random locations and interstimulus intervals (with a minimum of 100 ms). Within a trial, stimuli never occurred twice in the same box.

After 20 s, an empty grid appeared on the screen, marking the start of the retrieval phase. Here, participants were required to report the location and order of the apples that appeared during the encoding phase. They could do this by pointing to the corresponding boxes on the grid or by using verbal descriptions, such as "blue boat" or "black train." The experimenter recorded all responses by clicking the boxes in the exact order reported by the participant. The retrieval phase had no time limit, and participants were allowed to make any changes to their responses if they wished. The retrieval phase ended once the participant had assigned a location and order for each of the stimuli and confirmed that this was their final answer. Scoring was based on the location (e.g., bottom left), order (e.g., second), and sequence (correct predecessor or successor) of the apples, awarding a maximum of three points for each response. Feedback provided after each trial included the percentage correct score as well as a list of the correct/incorrect responses.

The VSWM task was preceded by an adaptive testing procedure in which the difficulty of the task was adjusted until an average percentage correct score of approximately 75% was reached. Starting at 5 stimuli per trial, the average percentage correct score was evaluated based on three-trial blocks, increasing or decreasing the working memory load by adding or removing 1 stimulus until performance converged around 75%. Due to differences in individual working memory capacity, the final number of stimuli used to perform the VSWM task ranged from 4 to 12. To ensure that all participants received comparable levels of practice, the minimum number of adaptive testing blocks was set to 4 (even if your performance converged to 75% in the second block of the adaptive testing, participants were required to perform an additional 2 blocks). After the adaptive testing, the number of stimuli stayed fixed throughout the experiment.

Postural control was assessed using the NeuroCom Balance Master^®^ (NeuroCom International Inc., Clackamas, OR), comprising a 46 × 46 cm AMTI dual force plate. The position of the body’s CoP was calculated for each centisecond (100 Hz sampling frequency) by recording lateral, vertical, and horizontal ground reaction forces. Participants stood on the force platform wearing a safety harness with their feet shoulder width apart and the arms hanging down. They were instructed to stand as still as possible once the verbal starting cue was provided (“ready, start”). In the stable stance condition, the force platform and cabin surround stayed fixed. During sway-referenced trials, stability was challenged by rotating both the force platform and cabin surround within the sagittal plane in direct proportion to the participant’s CoP motion^53^. Trials took 24 s and information regarding the difficulty manipulation was provided before each trial. Evaluation of the balance performance included summary statistics as well as stabilogram diffusion analysis (see “Preprocessing” section).

Postural control trials were preceded by a warm-up protocol specifically designed to make all individuals comfortable with the combined sway-referenced vision and support manipulations. Therefore, the warm-up included 15 s trials to make the participants familiar with the components (sway-referenced vision, sway-referenced support) as well as the combined sway-referencing used in the experimental protocol. If participants were unable or anxious to perform the combined sway-referencing at the default level of sway gain (with the visual surround and support surface matching the participants sway^54^), the gain was adjusted to the individual’s capacity. As a consequence, the sway gain was lowered from 1 (default level) to 0.75 in 6 middle-aged and 9 older adults and to 0.5 in 5 older adults.

The RT task required simple, speeded reactions to 8 tone beeps, (660 Hz, 150 ms) presented at pseudorandom interstimulus intervals (2000 ms, 2500 ms, 3000 ms) via headphones. Participants were asked to click the left mouse button as soon as possible each time they heard a beep. The task was performed using a custom-written script in Matlab, with each trial lasting 20 s. Feedback regarding the participant’s average RT and incorrect responses was provided after each trial. Incorrect responses were defined as responses faster than 100 ms or slower than 2000 ms (< 4% of responses). The RT trials were preceded by a warm-up trial. The outcome variable for data analysis included the mean RT of all correct responses.

Apart from all tasks being performed in isolation (single-task context), the VSWM task was combined with either the postural control or the RT task (dual-task contexts). The VSWM and RT tasks performed in isolation (single-task) as well as their combination (dual-task) were performed seated. The postural control single-task, as well as the combination of the VSWM and postural control tasks, were performed while standing. Dual-task contexts were designed such that only the encoding phase of the VSWM task overlapped with the RT/postural control task. Consequently, in the VSWM-postural control combination, postural control measurement (24 s) commenced before the VSWM encoding (20 s). This ensured participants achieved a stable posture prior to the presentation of the first visual stimulus, which was especially important in the sway-referenced condition.

Single-task postural control assessment included a secondary task designed to make single and dual-task conditions comparable in terms of head movement and visual fixations. This control task consisted of a simplified version of the VSWM task, in which one or two stimuli had a different color (i.e., yellow). Participants were asked to remember the last item which had a different color. All participants successfully performed the control task without errors. When performing the postural control task (single-task or combined with the VSWM task), the built-in Planar PL1500M 15″ XGA display was used (part of the NeuroCom Balance Master^®^) to present the visual stimuli. The Dell laptop screen was adjusted to match the resolution and aspect ratios of this built-in display in order to maximally control for any discrepancies between single- and dual-task conditions.

### Procedure

All experimental data was collected during one session. Single-task stable stance, sway-referenced and RT tasks were assessed in 2 two-trial blocks, one in the beginning and one at the end of the session. Single-task VSWM performance was also assessed in the beginning and at the end, but included three-trial blocks and an additional assessment in the middle of the session. Dual-task combinations (i.e., VSWM-stable stance, VSWM-sway-referenced, VSWM-RT) were assessed in 2 two-trial blocks. One block occurred after the initial single-task assessments, and the other block preceded the final single-task assessments. The goal of this block design was to minimize fatigue and practice effects.

### Pre-processing

All preprocessing and statistics were performed using R 4.1.2 (R Core Team, 2021). For the RT task, incorrect responses and responses to the first beep of each trial were excluded. For CoP data, a fourth-order low-pass Butterworth filter of 13 Hz was applied^55^. To account for small asynchronies in timing between the postural control and VSWM tasks, the first and last second of the CoP data were excluded from data analysis. The ellipse area including 95% of the CoP datapoints was used as a traditional postural control summary measure^56^. In addition, stabilogram diffusion analysis (SDA) was used to quantify component processes of postural control^38^. The methodology used for calculating the SDA parameters followed the approach outlined in Van Humbeeck et al.^27^. In summary, stabilogram diffusion analysis goes beyond traditional parameters that simply measure total displacement (the sum of displacements between all data points at the smallest time intervals). Instead, it considers the displacement of data points across all possible time intervals ranging from 10 ms (smallest time interval at 100 Hz sample rate) to 10 s. This more comprehensive approach involves calculating the average squared displacement for each time interval. The resulting graph typically shows that CoP dynamics follow a distinct two-part trend: a steep initial rise in mean squared displacement at short time intervals (representing short-term diffusion) that transitions to a much shallower slope at longer time intervals (representing long-term diffusion). This transition typically occurs around the critical time interval of 500–1000 ms. In order to determine the transition from short- to long-term control processes, the Segmented function of the Segmented package in R was used^57^.

### Statistical analysis

Prior to statistical analyses we checked distributions of dependent variables using the Box-Cox method in R. The procedure advised logarithmic transformations to obtain normality for reaction times and all postural control variables but the long-term diffusion coefficient, where a negative power transformation (λ = 0, ɣ = 0.2) was required. First, we evaluated whether we were successful in individually adjusting the single-task demands of the VSWM task by examining the age-related differences in VSWM load and related percentage correct scores across age groups. Then, linear mixed-effects models (LMMs, lme4 packages^58,59^) were used to explore how multitasking affected performance on each task (VSWM, reaction time, postural control) while considering potential interactions between age and task difficulty. Additionally, separate analyses for each of the stabilogram diffusion parameters in AP- and ML-directions were conducted to investigate the impact of multitasking on specific aspects of postural control. Lastly, to answer our research questions with regard to age-related performance differences in the different task combination, a final set of analyses was performed in which we used Z-transformed performance measures to incorporate performances of both combinations in to one statistical model.

All base LMMs included fixed effects for context (single-task, dual-task), age group (children, young adults, middle-aged adults, older adults), and subject (intercept) as a random factor. The context comparison was assessed as part of a prespecified contrast in which we compared the average response across single-task blocks with the average response across dual-task blocks. Other block comparisons, specifically those involving the first and last single-task block as well as the first and last dual-task block, were considered factors of no-interest. For the models involving VSWM, we contrasted average responses across tasks, as they contained different numbers of single- and dual-task blocks.

Age-group comparisons were assessed using 4 prespecified contrasts. The children-young adults contrast compared the mean of all children with young adults; the adult-age contrast compared young adults with the mean of middle-aged and older adults; the late-adulthood contrast compared middle-aged and older adults. Finally, we specified the extreme-age group contrast as a comparison of children and older adults. When comparing costs between different combinations of tasks we used z-scored performance outcomes in VSWM, postural-control, and RT tasks as levels of the additional fixed factor “measure”. To assess additional developmental effects within age groups we included exact age (centered) as a nested factor within age groups. Sex and norm-referenced BMI (the latter two as nested within age-groups) were included as covariates of no-interest along with their interactions.

To obtain the most parsimonious model fit (i.e., fewest necessary variables), we followed the parsimonious model selection approach proposed^60^. We performed a stepwise backwards exclusion procedure, removing non-significant covariates and interactions at p > 0.05. In a second step, we tested whether expanding the random effect structure by allowing subject by context and /or measure interactions improved the model fit. Post-hoc tests were performed using Bonferroni adjusted t-tests. Results regarding model selection and covariates are addressed in the Supplementary Notes.

### Supplementary Information

Supplementary Information.

## Data Availability

The datasets analyzed during the current study are available from the corresponding author on reasonable request.
