# Supplementary material for: Multitasking across the lifespan in different task contexts
Source: Sci Rep. 2024 May 23;14:11817. doi: 10.1038/s41598-024-61859-w (PMC11116417; doi:10.1038/s41598-024-61859-w)
Supplement: Supplementary file 1 — Supplementary Information. [file 41598_2024_61859_MOESM1_ESM.docx]

# Supplementary Notes

## Model Selection

For the models involving postural control, nested effects of sex improved the model fit in all parameters (χ2(4)≥ 10.370, p<0.035) except from the AP long-term diffusion coefficient (χ2(4) = 2.991, p=0.559). Nested age contributed to the model fit in the ellipse area, ML short-term diffusion and ML critical mean squared displacement parameters (χ2(4)≥ 10.330, p<0.035). Norm-referenced BMI only contributed to the model of the ML critical time interval (χ2(4) = 15.528, p=0.004). A sex by task block interaction improved the model fit in the AP critical time interval (χ2(12) = 21.228, p=0.047). For VSWM task performance, model selection was performed separately for each of the multitasking settings (figure 2 main text). Nested effects of sex and its interaction with context contributed to the VSWM-RT setting (χ2(4)= 10.892, p=0.028). Neither nested sex nor nested age effects contributed to the VSWM-postural control task setting (χ2(4)= 5.112 , p=0.276; χ2(4)= 4.031, p=0.402). For the reaction time task, model selection showed that nested effects of sex, nested effects of age, and both of their interactions with the task block contributed to the model (χ2(12)= 21.940 p=0.038; χ2(12)= 44.466, p<0.001).
